# On Writing Prescriptions

**Published:** 1853-03

**Authors:** 


From the Medical Recorder.

*On Writing Prescriptions.*

The following remarks were suggested to us upon observing the subjoined Prescription among the transactions of the Berks County Medical Society, as presented to the Central Society of the State of Pennsylvania. Special attention was invited to this report, as the most valuable one of the year, by the Central Publishing Committee:

| | |
|---|---|
| ℞—Hydrochlor. ammoniæ, | ʒij |
| Magnesiæ Sulph., | ʒvj |
| Ant et Potass. Tart., | gr. i |
| Syr. Acid. Citrici, | ℥ss |
| Aquæ, | ℥iv |

The prescription was administered in scarlatina for the purpose of producing diaphoresis, and, as we suppose, general relaxation of the system—at least, the ingredients, separately taken, are calculated to produce these effects. But the merest tyro in pharmacy must perceive that the whole is a forrago of incompatible substances; the first two would mutually decompose one another—a arge portion of the Epsom Salts being converted into the Chloride of Magnesium, and all the Sal Ammoniac into Sulphate of Ammonia. Then, again, the last two would, by their reaction, convert the Tartrate into the Citrate of Antimony, and this would still be incompatible with the resultants of the first decomposition. What might be the ultimate resultants when all the decompositions and recompositions caused by compounding these discordant elements had taken place, it would puzzle a much more practiced chemist than ourselves to determine—certainly nothing which was present to the mind of the Physician when he made the prescription.

The effect intended, we are informed, was produced, probably from the presence of some salt of antimony—nearly all the salts of that metal having a diaphoretic virtue; and probably the whole mixture would have been more efficacious if everything had been left out except the antimonial and the water.

Two inferences shall here be drawn from the premises—

Firstly. Physicians ought to know *a little* about chemistry. *A very little indeed*, would be necessary to preclude such glaring blunders as the above.

Certainly, we have a Professor of Chemistry attached to every one of our Medical Colleges, who teaches the students through two sessions, and examines them upon their proficiency afterwards. Here is the apparatus for securing the Profession against incompetency of this character. It is equally certain that, wherever the defect may be in its working, the apparatus does not produce the objects intended—that by far the majority of the countless hordes of graduates who are every year issuing from our universities, know not chemistry enough to secure themselves from becoming the laughing stock of the first intelligent apothecary's apprentice to whom they may send an incompatible prescription. But this is the business of those who have the power to prevent it. Let us proceed to infer

Secondly. Whether they understand chemistry or not, physicians, especially young ones, would do well to simplify their prescriptions. Those who do not know chemistry, will thereby avoid exposing their ignorance, not to mention the risk of neutralizing one ingredient by another incompatible with it; while those who do, will be able to study their own practice in medication, with some prospect of knowing which of their remedies has produced a given effect. We think it will be found that a gradual simplification of prescriptions has been observing a progress exactly parallel with that of enlightenment in medical science. In the recipes preserved from the practice of 200 years ago, thirty ingredients was a moderate number, and we find among them the brains of rats, the incinerated entrails of cats, the testicles of Guinea pigs, &c., &c.; and in the practice of even ten years ago, from six to ten is a moderate number of items; while the most successful practitioners of the present day have been more disposed to study the specific operation of one medicine at a time, than to confuse their own observations by combining half an apothecary's shop in a prescription. D. F. W.

